# Juvenile myoclonic epilepsy as a spectrum disorder: mechanisms of drug resistance and precision management

**DOI:** 10.3389/fneur.2026.1802052

**Published:** 2026-04-16

**Authors:** Xiaoping Liu, Meizhen Sun, Xiaoping Du

**Affiliations:** 1 First Clinical Medical Department, Shanxi Medical University, Taiyuan, China; 2 Department of Neurology, First Hospital of Shanxi Medical University, Taiyuan, China; 3 Neurosurgery Center, Department of Functional Neurosurgery, The National Key Clinical Specialty, Guangdong Provincial Key Laboratory on Brain Function Repair and Regeneration, Zhujiang Hospital Institute for Brain Science and Intelligence, Zhujiang Hospital, Southern Medical University, Guangzhou, China

**Keywords:** brain networks, drug resistance, EEG, juvenile myoclonic epilepsy, neuromodulation, precision medicine, spectrum disorder

## Abstract

Juvenile myoclonic epilepsy (JME) has traditionally been regarded as a homogeneous and pharmacoresponsive epilepsy syndrome. However, a substantial proportion of patients do not achieve sustained seizure freedom, challenging this concept of benignity. Increasing evidence suggests that JME represents a spectrum disorder characterized by clinical, electrophysiological, and neurobiological heterogeneity. In this review, we re-examine drug resistance (DR) in JME from a spectrum-based and systems-level perspective. We distinguish true DR from pseudo-resistance, highlighting the contribution of modifiable factors (e.g., misdiagnosis, inappropriate ASM selection, lifestyle influences, and poor adherence). We further synthesize multilevel determinants of treatment response, encompassing clinical features, EEG markers, genetic susceptibility, and large-scale brain network dysfunction. Based on these insights, we propose a dynamic model of treatment responsiveness and discuss its implications for individualized management. Finally, we outline future directions toward integrated, biomarker-driven, and precision medicine approaches in JME. This framework aims to refine risk stratification and support more individualized and mechanism-informed therapeutic decision-making in this common but heterogeneous epilepsy syndrome.

## 1 Introduction

Juvenile myoclonic epilepsy (JME) is a common idiopathic generalized epilepsy, accounting for 5–10% of all epilepsies and up to 18% of generalized epilepsies (1). Traditionally regarded as benign and pharmacologically responsive, valproic acid achieves seizure control in most patients (2). Real-world evidence indicates that ~36.6% of patients do not achieve sustained seizure freedom despite adequate antiseizure medication (ASM) therapy (3). These patients may experience persistent seizures despite treatment, along with cognitive dysfunction, psychiatric comorbidities, and impaired psychosocial outcomes, often described as drug resistance (DR) in JME. Whether DR in JME represents a distinct entity or reflects dynamic outcomes influenced by heterogeneous, modifiable factors remains debated.

The International League Against Epilepsy (ILAE) defines drug-resistant epilepsy (DRE) as failure of two adequate ASM trials; however, this definition is context-sensitive and influenced by non-biological modifiable factors, including diagnostic delay, inappropriate drug selection, poor adherence, and lifestyle factors (4). In this review, DR is conceptualized as a continuum, extending beyond this operational definition, encompassing dynamic and potentially reversible influences on treatment response. However, a unified framework integrating these dimensions to explain variability in treatment response in JME remains lacking.

JME is increasingly recognized as a spectrum disorder. Beyond the classical triad of myoclonic, bilateral tonic–clonic seizures (BTCS), and absence seizures, patients exhibit variable reflex and photosensitive features, cognitive profiles, and developmental trajectories, sometimes overlapping with childhood absence epilepsy (5). Mechanistic studies highlight JME as a disorder of large-scale brain networks, particularly cortico-thalamo-cortical circuits, where altered connectivity, network instability, and polygenic influences converge to shape seizure susceptibility and pharmacoresponsiveness (6–11).

In this review, we adopt a spectrum- and systems-level approach to integrate clinical, electrophysiological, genetic, and network-level evidence to inform precision medicine–oriented management strategies.

## 2 JME as a spectrum disorder

### 2.1 From classical syndrome to spectrum concept

Initially, JME was described as a relatively homogeneous syndrome, characterized by myoclonic jerks on awakening, often accompanied by BTCS and occasionally absence seizures (12). Early ILAE classifications (1989) emphasized adolescent onset, generalized spike–wave discharges, photosensitivity, and favorable ASM response (13).

Subsequent ILAE revisions through 2022 have broadened diagnostic boundaries, recognizing wider onset ages, variable EEG patterns, and greater clinical heterogeneity (14). Family studies and long-term follow-up demonstrate overlapping subphenotypes with distinct courses and outcomes (15). Collectively, these findings support a spectrum-based model encompassing variability in seizure types, triggers, cognitive profiles, and disease trajectories (5).

### 2.2 Developmental continuum and clinical heterogeneity

JME shares features with other idiopathic generalized epilepsies, particularly childhood absence epilepsy (CAE). In some patients, absence seizures in childhood evolve into myoclonic and BTCS in adolescence (5, 15, 16). This trajectory reflects dynamic changes in brain maturation, network connectivity, and cortical excitability, contributing to variability in disease severity and treatment response.

Phenotypic heterogeneity is further evident in seizure composition, reflex and photosensitive features, motor, cognitive, and psychiatric traits (3, 15, 17–22). In addition, diagnostic and methodological factors—including absence of sleep EEG and other modifiable factors—further influence apparent treatment response (3, 15, 17, 23–27).

### 2.3 Implications for DR

Within this spectrum framework, DR in JME is best conceptualized as an emergent outcome of interacting factors rather than a discrete disease subtype. Biological susceptibility, including genetic predisposition and network instability, interacts with phenotypic complexity—such as multiple seizure types and overlapping subsyndromes—as well as diagnostic and management-related factors, including delayed recognition, misdiagnosis, and suboptimal treatment (4, 6–11, 15, 26–28).

Accordingly, pseudo-resistance (apparent DR) in JME should be understood as a dynamic and potentially reversible state along a continuum, shaped by both intrinsic neurobiological mechanisms and modifiable external influences. This perspective informs the following re-evaluation of DR in JME.

## 3 Rethinking DR in JME

### 3.1 Conceptual and diagnostic challenges in DR

The ILAE defines drug resistance as failure of two adequate ASM trials (4). In JME, this definition has traditionally been interpreted as suggesting a discrete subgroup. However, substantial heterogeneity in clinical presentation, developmental trajectory, and neurobiology supports a continuum model of DR, in which treatment response reflects the interaction of intrinsic susceptibility and modifiable external factors (5, 15, 29).

Distinguishing true (intrinsic) DR from pseudo-resistance is therefore critical. Misdiagnosis—particularly as focal epilepsy—often leads to inappropriate use of sodium channel–blocking ASMs, which may aggravate seizures and mimic treatment failure (15, 26, 27). Additional contributors include modifiable factors such as sleep deprivation and alcohol use (3, 17).

Importantly, many patients initially classified as having DR achieve seizure control after diagnostic reassessment and treatment optimization, underscoring the clinical relevance of pseudo-resistance (30, 31). Failure to identify these reversible contributors may lead to overestimation of true, biologically driven DR.

### 3.2 Biological determinants of DR

Persistent seizures despite optimized treatment likely reflect intrinsic, biologically driven DR.

Clinically, early onset, multiple seizure types, and a history of absence seizures are associated with poorer outcomes (3, 17, 28, 32). At the systems level, JME involves dysfunction of cortico-thalamo-cortical networks, with altered connectivity and reduced network stability affecting both seizure generation and treatment response (7–11).

Genetic susceptibility further modulates these processes. As a polygenic disorder, JME involves variation in neuronal excitability, synaptic transmission, and network organization, contributing to interindividual variability in pharmacoresponsiveness (6).

Together, these factors define a spectrum of treatment responsiveness by underlying neurobiology, rather than a uniform DR state.

### 3.3 Dynamic mode

**Figure 1:**
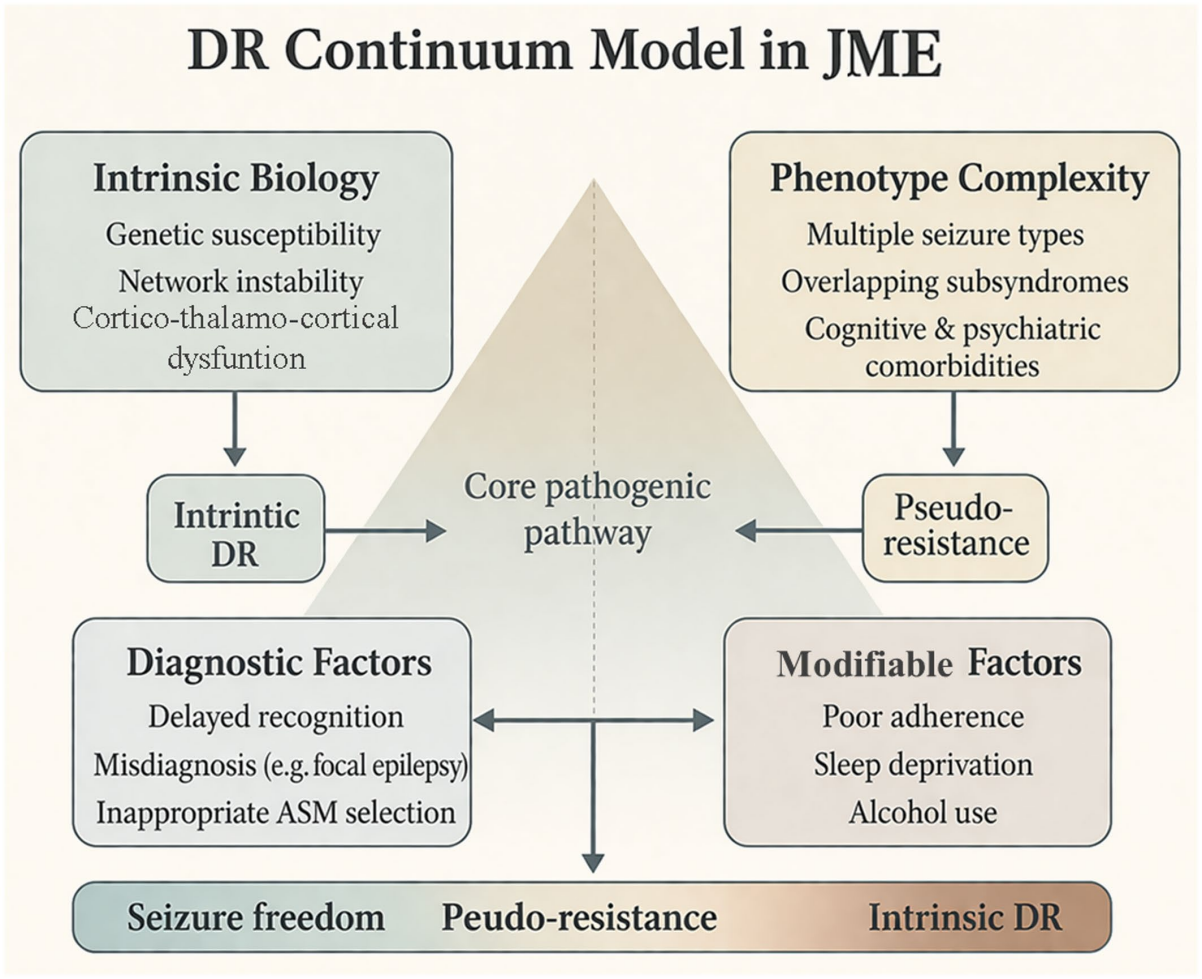
The continuum model of drug resistance in juvenile myoclonic epilepsy. Drug resistance (DR) arises from the interaction between intrinsic biological susceptibility—such as genetic predisposition and network instability—and phenotypic complexity, including multiple seizure types and overlapping subsyndromes. Pseudo-resistance emerges from modifiable diagnostic and management-related factors, including delayed recognition, misdiagnosis, inappropriate antiseizure medication (ASM) selection, poor adherence, sleep deprivation, and alcohol use. These dimensions intersect with the core cortico-thalamo-cortical pathogenic network, shaping treatment outcomes along a continuum from seizure freedom to intrinsic DR. JME, juvenile myoclonic epilepsy.

Treatment response in JME is dynamic and exists along a continuum from sustained seizure freedom to persistent seizures (33). As illustrated in Figure 1, DR arises from the interaction between intrinsic biological susceptibility—such as genetic predisposition and network instability—and phenotypic complexity, including multiple seizure types and overlapping subsyndromes (17, 28, 34).

This state is not fixed and may evolve over time with changes in modifiable factors and disease progression, consistent with network-based models of epilepsy (35). Accordingly, the designation of DR in JME should be applied cautiously, as it may obscure both heterogeneity and potential reversibility.

Clinically, this framework emphasizes the need to systematically exclude reversible factors, integrate clinical and neurobiological information for risk stratification, and adopt individualized, mechanism-informed treatment strategies. It also provides the conceptual foundation for subsequent discussion of multilevel predictors and precision medicine approaches in JME.

## 4 Multilevel predictors of DR in JME

Treatment response in JME is shaped by multiple interrelated factors spanning clinical, genetic, and network domains. Key predictors across these dimensions are summarized in Table 1, providing a concise reference for risk stratification.

**Table 1:** Multilevel predictors of treatment response in juvenile myoclonic epilepsy.

Category	Predictor	Description	Prognostic implication	Key references
Clinical factors	Multiple seizure types	Coexistence of myoclonic seizures, generalized tonic–clonic seizure, and absence seizures	Associated with poorer seizure control and increased risk of persistent seizures	(3, 17, 28, 32)
	Absence seizures	History or presence of absence seizures	Negative prognostic factor; reflects broader network involvement	(28, 32)
	Early age at onset	Onset in early adolescence or childhood	May indicate more severe or widespread disease	(3, 17)
	Long disease duration	Prolonged untreated or active epilepsy	Associated with reduced likelihood of remission	(3, 17)
	Modifiable factors	Sleep deprivation, alcohol use, poor adherence, misdiagnosis, suboptimal treatment	Major modulators of seizure occurrence; may mimic drug resistance (pseudo-resistance)	(3, 17)
EEG features	High epileptiform discharge burden	Frequent generalized spike–wave or polyspike–wave discharges	Associated with increased network excitability and poorer control	(23, 24)
	Persistent EEG abnormalities	Continued epileptiform activity despite treatment	May indicate ongoing network instability	(23, 24)
	Photosensitivity	Photoparoxysmal response on EEG	Reflects specific network vulnerability; prognostic value variable	(19, 20)
	Sleep EEG abnormalities	Enhanced epileptiform discharges during sleep or sleep deprivation	Improves detection of disease severity; may relate to outcomes	(23, 24)
Genetic/molecular factors	Polygenic susceptibility	Variants affecting ion channels and synaptic transmission	Contributes to interindividual variability in treatment response	(6)
	Neurotransmitter system alterations	GABAergic and glutamatergic dysfunction	May influence seizure threshold and ASM response	(6)
	Subsyndrome-related genetic background	Genetic heterogeneity underlying JME subtypes	Indirectly associated with prognosis and treatment response	(15)
Network/imaging factors	Cortico–thalamo–cortical dysfunction	Abnormal connectivity within thalamocortical circuits	Core mechanism of seizure generation and persistence	(7–11)
	DMN disruption	Altered functional connectivity in DMN	Associated with impaired network stability	(7–11)
	Widespread network abnormalities	Diffuse structural or functional alterations	May predict reduced responsiveness to pharmacotherapy	(7–11)
	Network instability (functional connectivity)	Altered synchronization and network dynamics	Potential biomarker of treatment resistance	(7–11)

EEG, electroencephalogram; GABA, gamma-aminobutyric acid; ASM, anti-seizure medications; JME, juvenile myoclonic epilepsy; DMN, default mode network.

### 4.1 Clinical predictors

Within a spectrum-based framework, treatment response in JME is primarily shaped by clinical features and electrophysiological markers.

Seizure type composition is the most robust clinical predictor. Patients with multiple seizure types—particularly the coexistence of myoclonic, BTCS, and absence seizures—consistently show less favorable outcomes, whereas isolated myoclonic seizures are associated with better seizure control (3, 17, 28, 32). Early onset, longer disease duration, and a history of absence seizures further indicate increased disease complexity and reduced likelihood of sustained seizure freedom (3, 17, 28, 32). Lifestyle-related factors, including sleep deprivation, alcohol use, and poor adherence, also significantly influence seizure control and may contribute to pseudo-resistance (3, 17).

EEG provides objective indices of network excitability. Higher epileptiform burden, increased discharge frequency, and persistence of generalized spike- or polyspike-wave activity during treatment is associated with a reduced likelihood of sustained seizure freedom (23, 24). Photosensitivity and reflex EEG responses may reflect specific network vulnerabilities, although their prognostic value remains inconsistent (19, 20). Sleep EEG enhances detection of epileptiform activity and may better capture underlying network instability.

### 4.2 Genetic and network-level determinants

Genetic susceptibility and large-scale network organization represent key biological substrates of treatment response.

JME is a polygenic disorder involving variants that influence neuronal excitability, synaptic transmission, and network regulation (6). Although no single genetic marker reliably predicts treatment outcome, genetic heterogeneity contributes to interindividual differences in pharmacoresponsiveness and may underlie distinct subphenotypes within the JME spectrum (15).

At the systems level, neuroimaging studies consistently demonstrate abnormalities in cortico-thalamo-cortical circuits and disruptions in large-scale networks, including the default mode network (DMN) (7–11). Greater extent of network dysfunction is associated with reduced likelihood of complete seizure control. Emerging approaches, such as functional connectivity and graph theoretical analyses, offer potential network-based biomarkers, although their clinical utility remains to be established.

### 4.3 Integration and risk stratification

The clinical relevance of these predictors lies in their integration into multidimensional risk frameworks.

Treatment responsiveness in JME is best conceptualized as a continuum. Patients with simpler clinical phenotypes and lower epileptiform burden are more likely to achieve seizure freedom, whereas those with greater clinical and network complexity are at increased risk of incomplete control (28, 36, 37). Importantly, persistent seizures do not necessarily indicate intrinsic DR, as modifiable factors frequently contribute (28, 38).

Table 1 provides a structured summary of these multilevel predictors, enabling clinicians to synthesize complex data for individualized risk assessment and tailored management decisions. This integrated perspective underscores that treatment responsiveness exists along a continuum, rather than representing a binary drug-resistant state.

## 5 Therapeutic strategies in JME

### 5.1 Pharmacological strategies

Pharmacological therapy remains the cornerstone of JME management and should be individualized according to seizure phenotype and patient-specific factors, with ongoing reassessment to optimize long-term outcomes.

Valproate is the most effective first-line ASM, demonstrating broad-spectrum efficacy across seizure types (1, 2), likely through combined effects on inhibitory neurotransmission and network stabilization. However, safety concerns—particularly in women of childbearing potential—necessitate consideration of alternative ASMs, including levetiracetam and lamotrigine.

The selection of combination therapy should be individualized based on seizure type. Levetiracetam is widely prescribed owing to its favorable tolerability and ease of use, whereas lamotrigine may exacerbate myoclonic seizures in a subset of patients. Brivaracetam represents a viable alternative for myoclonic seizures (39). For bilateral tonic–clonic seizures, levetiracetam and lamotrigine are commonly utilized (40), with adjunctive options such as topiramate (41), and perampanel (42) providing additional therapeutic benefit. Ethosuximide may be considered as adjunctive therapy for absence seizures (42).

Importantly, multicenter retrospective investigations indicate that approximately 70% of patients with DR achieve seizure freedom following pharmacotherapy optimization, with the combination of valproate and lamotrigine associated with the highest therapeutic efficacy (43).

Conversely, sodium channel–blocking ASMs (e.g., carbamazepine, phenytoin, oxcarbazepine) should be avoided, as they may exacerbate seizures and contribute to pseudo-resistance, particularly in cases of misdiagnosis (15, 26, 27).

Overall, optimal pharmacological management in JME requires precise alignment between drug mechanisms and seizure phenotype, supported by iterative treatment adjustment to improve long-term outcomes.

### 5.2 Network-based and precision approaches

For patients with persistent seizures despite optimized pharmacotherapy, treatment should shift toward network-oriented and individualized strategies, integrating clinical, electrophysiological, and imaging data.

Neuromodulation offers a promising complementary approach by directly targeting distributed epileptic networks. Evidence indicates that vagus nerve stimulation (VNS) can reduce seizure frequency and improve quality of life, although data specifically in JME remain limited (44–51). Similarly, deep brain stimulation (DBS), particularly targeting centromedian and anterior thalamic nuclei, offers more focal modulation of thalamocortical circuits, with emerging evidence of efficacy in generalized epilepsies (46, 52–59). Key parameters for patient selection, efficacy outcomes, and safety considerations for these neuromodulation strategies are synthesized in Table 2, providing a practical reference for clinicians managing drug-resistant JME. This framework underscores the role of neuromodulation as part of a precision medicine approach, in which treatment decisions are guided by the integration of multimodal patient data.

**Table 2:** Neuromodulation outcomes of drug resistance juvenile myoclonic epilepsy.

Category	Targets	Stimulation parameters	Outcome	Key references
i-VNS	Left vagus nerve	Stimulation:250-500 μs pulse width, frequency 20–30 Hz, intensity 1.0–3.5 mA, 30–60 s on/5–10 min off	Five out of seven patients with juvenile myoclonic epilepsy were responders.	(49, 50)
TA-VNS	ABVN	Stimulation: 0.2 s pulse width, frequency >30 Hz (stably ≤50 Hz, below discomfort threshold), 4 times/d, 30 min/session	The overall efficacy rate was 61.54%	(48, 51)
DBS	CM	Medium and high frequencies (60–145 Hz) with 90–450 μs pulse width and 1–10 V intensity	Over 80% of patients were considered responders	(52, 54–56)
	ANT	High-frequency stimulation between 90 and 200 Hz with 90–160 μs pulse width and 1.5–10 V intensity	By 2 years, there was a 56% median percent reduction in seizure frequency	(52, 57)
	STN	High-frequency stimulation	Motor seizure frequency decreased by 87.5%	(58, 59)

i-VNS, invasive vagus nerve stimulation; TA-VNS, transcutaneous auricular vagus nerve stimulation; ABVN, auricular branch of the vagus nerve; DBS, Deep brain stimulation; CM, centromedian; ANT, anterior nucleus of thalamus; STN, Subthalamic nucleus.

**Figure 2:**
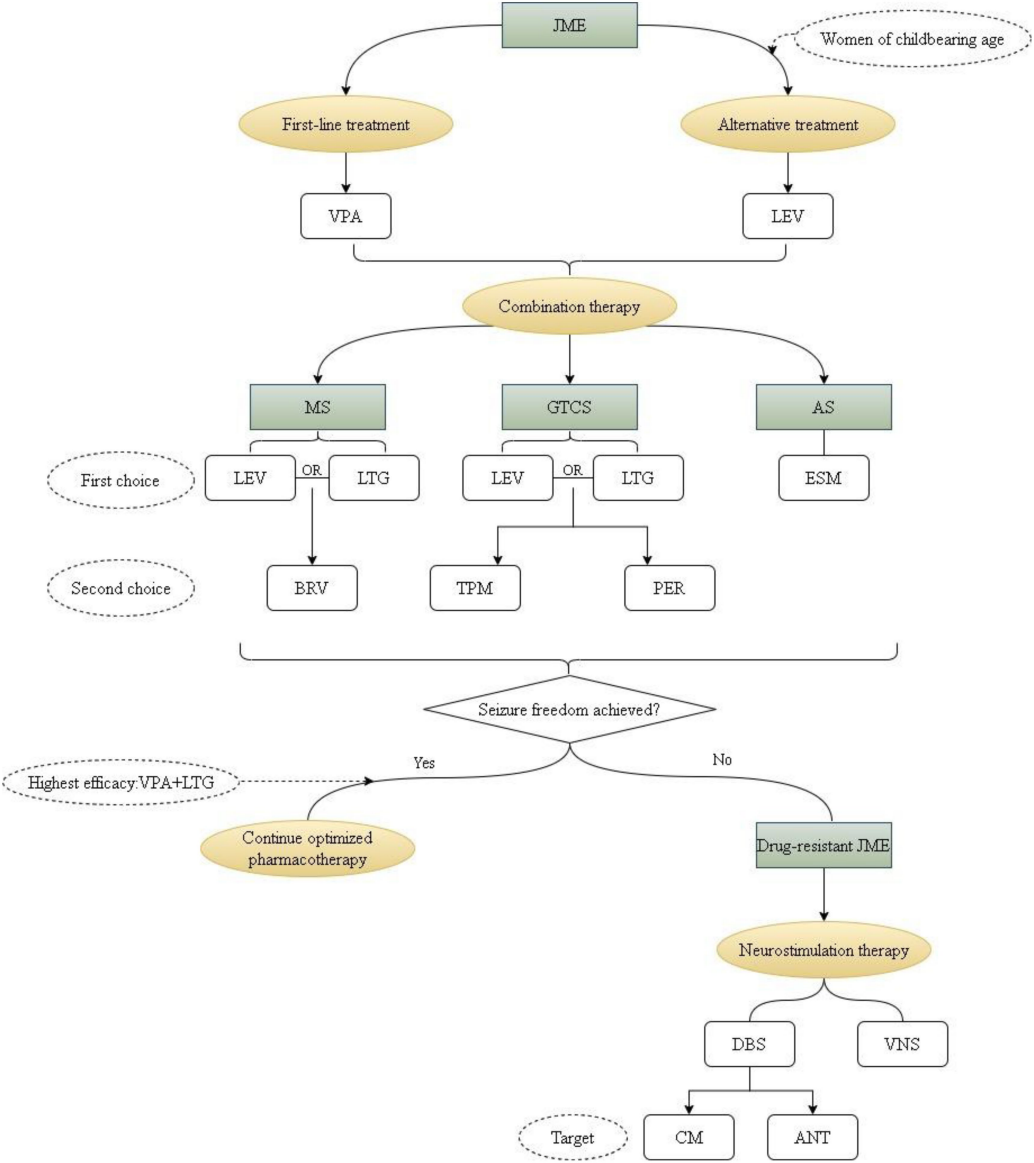
Therapeutic strategies in juvenile myoclonic epilepsy. Management begins with individualized pharmacological therapy based on seizure phenotype and patient-specific factors. Valproate remains the most effective first-line antiseizure medication (ASM), although alternative agents such as levetiracetam and lamotrigine are frequently used when contraindications exist. Treatment should be further tailored according to seizure type, with adjunctive options introduced as needed. In patients with persistent seizures despite optimized therapy, evaluation of pseudo-resistance and modifiable factors is essential. Selected patients may benefit from network-based interventions, including vagus nerve stimulation (VNS) and deep brain stimulation (DBS). Overall, treatment outcomes span a continuum from seizure freedom to drug resistance (DR). JME, juvenile myoclonic epilepsy; VPA, valproate; LEV, levetiracetam; LTG, lamotrigine; ESM, ethosuximide; BRV, brivaracetam; TPM, topiramate; PER, perampanel; CM, centromedian; ANT, anterior nucleus of thalamus.

To operationalize individualized management, a precision therapy algorithm has been developed, illustrated in Figure 2. This algorithm emphasizes exclusion of reversible factors, stepwise optimization of therapy, and incorporation of neuromodulation within multidisciplinary care pathways. By linking patient-specific clinical, electrophysiological, and network-level information, it supports adaptive treatment adjustments and highlights patients most likely to benefit from neuromodulation.

Despite these advances, limitations remain, including limited syndrome-specific evidence and lack of standardized protocols, underscoring the need for prospective studies to identify predictors of response and refine patient selection strategies.

## 6 Outcomes beyond seizures

Although seizure control remains the primary therapeutic goal in JME, many patients experience persistent non-seizure-related impairments. Cognitive deficits—particularly in attention, working memory, and executive function—are reported even in individuals with well-controlled seizures (2, 20). Psychiatric comorbidities, including anxiety and depression, further impair quality of life and complicate management (5, 21, 22).

Beyond individual morbidity, JME imposes substantial social consequences, with reduced educational attainment, occupational limitations, and restricted independence, highlighting that treatment success should extend beyond seizure freedom to encompass cognitive, psychiatric, and psychosocial outcomes.

## 7 Future directions

Future research in JME should move beyond single-domain frameworks toward integrated, multilevel models that combine clinical phenotypes, electrophysiological markers, genetic susceptibility, and network-level imaging to enable individualized risk stratification.

Advances in machine learning offer the ability to capture complex, non-linear interactions across these domains, with potential to refine disease subtyping, predict treatment responsiveness, and support data-driven clinical decision-making. In parallel, the identification of robust biomarkers—particularly EEG-based measures of synchronization and connectivity, alongside genetic signatures—may enable earlier recognition of high-risk patients and facilitate biomarker-guided therapy.

Ultimately, these developments support a shift from static, syndrome-based classification toward a dynamic, mechanism-informed framework. By linking phenotype, network dysfunction, and underlying biology, such an approach provides the foundation for precision medicine and more effective, individualized, and mechanism-informed management of JME. However, challenges remain, including data standardization, model interpretability, and clinical translation. Such integrative frameworks may enable a shift from reactive to predictive and preventive epilepsy care. Prospective, multicenter studies with standardized multimodal data collection will be essential to translate these models into clinical practice.
